# Supplementary material for: End Sequence Analysis Toolkit (ESAT) expands the extractable information from single-cell RNA-seq data
Source: Genome Res. 2016 Oct;26(10):1397–410. doi: 10.1101/gr.207902.116 (PMC5052061; doi:10.1101/gr.207902.116)
Supplement: Supplemental Material [file supp_gr.207902.116_Supp_Materials.tar.gz › ESAT-esat_v0.1/doc/html/classbroad_1_1pda_1_1geneexpression_1_1clustering_1_1_cluster.html]

ESAT: broad.pda.geneexpression.clustering.Cluster Class Reference


ESAT


- Main Page
- Related Pages
- Packages
- Classes
- Files

- Class List
- Class Index
- Class Hierarchy
- Class Members

All Classes Namespaces Files Functions Variables Enumerator Pages

- broad
- pda
- geneexpression
- clustering
- Cluster

Public Member Functions |
List of all members

broad.pda.geneexpression.clustering.Cluster Class Reference

Collaboration diagram for broad.pda.geneexpression.clustering.Cluster:

[legend]

|  |  |
| --- | --- |
| Public Member Functions | |
|  | Cluster (String col) |
|  | |
|  | Cluster (Collection< String > mem, String group) |
|  | |
| void | setScore (double score) |
|  | |
| double | getScore () |
|  | |
| boolean | hasSubClusters () |
|  | |
| void | addToCluster (String member) |
|  | |
| Pair< Cluster > | getSubclusters () |
|  | |
| Collection< String > | getMembers () |
|  | |
| void | setSubcluster (Cluster cluster1, Cluster cluster2) |
|  | |
| Cluster | getLeft () |
|  | |
| Cluster | getRight () |
|  | |
| List< String > | getOrdered () |
|  | |
| String | toString () |
|  | |

## Constructor & Destructor Documentation

|  |  |  |  |  |  |
| --- | --- | --- | --- | --- | --- |
| broad.pda.geneexpression.clustering.Cluster.Cluster | ( | String | *col* | ) |  |

|  |  |  |  |
| --- | --- | --- | --- |
| broad.pda.geneexpression.clustering.Cluster.Cluster | ( | Collection< String > | *mem*, |
|  |  | String | *group* |
|  | ) |  |  |

## Member Function Documentation

|  |  |  |  |  |  |
| --- | --- | --- | --- | --- | --- |
| void broad.pda.geneexpression.clustering.Cluster.addToCluster | ( | String | *member* | ) |  |

|  |  |  |  |  |
| --- | --- | --- | --- | --- |
| Cluster broad.pda.geneexpression.clustering.Cluster.getLeft | ( |  | ) |  |

|  |  |  |  |  |
| --- | --- | --- | --- | --- |
| Collection<String> broad.pda.geneexpression.clustering.Cluster.getMembers | ( |  | ) |  |

|  |  |  |  |  |
| --- | --- | --- | --- | --- |
| List<String> broad.pda.geneexpression.clustering.Cluster.getOrdered | ( |  | ) |  |

Here is the call graph for this function:

|  |  |  |  |  |
| --- | --- | --- | --- | --- |
| Cluster broad.pda.geneexpression.clustering.Cluster.getRight | ( |  | ) |  |

|  |  |  |  |  |
| --- | --- | --- | --- | --- |
| double broad.pda.geneexpression.clustering.Cluster.getScore | ( |  | ) |  |

|  |  |  |  |  |
| --- | --- | --- | --- | --- |
| Pair<Cluster> broad.pda.geneexpression.clustering.Cluster.getSubclusters | ( |  | ) |  |

|  |  |  |  |  |
| --- | --- | --- | --- | --- |
| boolean broad.pda.geneexpression.clustering.Cluster.hasSubClusters | ( |  | ) |  |

Here is the caller graph for this function:

|  |  |  |  |  |  |
| --- | --- | --- | --- | --- | --- |
| void broad.pda.geneexpression.clustering.Cluster.setScore | ( | double | *score* | ) |  |

|  |  |  |  |
| --- | --- | --- | --- |
| void broad.pda.geneexpression.clustering.Cluster.setSubcluster | ( | Cluster | *cluster1*, |
|  |  | Cluster | *cluster2* |
|  | ) |  |  |

|  |  |  |  |  |
| --- | --- | --- | --- | --- |
| String broad.pda.geneexpression.clustering.Cluster.toString | ( |  | ) |  |

---

The documentation for this class was generated from the following file:

- java/broad/pda/geneexpression/clustering/Cluster.java


---

Generated on Fri Aug 15 2014 15:11:00 for ESAT by  

 1.8.7
